# Threonine and tyrosine kinase (TTK) mRNA and protein expression in breast cancer; prognostic significance in the neoadjuvant setting

**DOI:** 10.1111/his.15399

**Published:** 2025-01-07

**Authors:** Abrar Ashi, Aeshah A Awaji, Jacquelyn Bond, Colin A Johnson, Abeer M Shaaban, Sandra M Bell

**Affiliations:** ^1^ Division of Molecular Medicine, Leeds Institute of Medical Research, St James's University Hospital University of Leeds Leeds UK; ^2^ Histopathology, St James's Institute for Oncology St James's University Hospital Leeds UK; ^3^ Histopathology and Cancer Sciences Queen Elizabeth Hospital Birmingham and University of Birmingham Birmingham UK

**Keywords:** breast cancer, MCPH1, neoadjuvant chemotherapy (NACT), TNBC, TTK

## Abstract

**Aims:**

Threonine and tyrosine kinase (TTK) is up‐regulated in triple‐negative breast cancer (TNBC), yet its expression in patients undergoing neoadjuvant chemotherapy (NACT) remains unexplored. This investigation aims to assess TTK protein expression in treatment‐naïve pre‐treatment cores and paired pre‐ and post‐NACT breast cancer (BC) cohorts, as well as its correlation with microcephaly 1 (MCPH1) protein expression.

**Methods and results:**

Transcriptomic data were sourced from the Gene Expression Omnibus microarray database for mRNA expression analysis. TTK protein expression was evaluated using immunohistochemistry staining, employing receiver operating characteristic curve analysis to determine an optimal TTK expression cut‐off point. The association between TTK expression, clinicopathological parameters and survival outcomes was examined. Additionally, MCPH1 protein expression was assessed in a pilot study. Analysis revealed a significantly elevated TTK mRNA expression in BC tissue compared to normal breast tissue, with high TTK mRNA levels predicting reduced overall survival. Notably, TTK protein expression increased significantly post‐NACT in a paired cohort. Conversely, decreased TTK protein expression pre‐NACT was correlated with improved overall survival.

**Conclusions:**

High TTK and low MCPH1 protein expression was significantly correlated, highlighting TTK's potential as a biomarker for BC and a therapeutic target for MCPH1‐deficient cancer cells.

## Graphical abstract

This study investigates threonine and tyrosine kinase (TTK) expression in pre‐ and post‐neoadjuvant chemotherapy breast cancer patients. TTK mRNA expression was elevated in breast cancer tissues, predicting reduced survival. Notably, TTK protein expression in neoadjuvant chemotherapy‐treated breast cancer patients was up‐regulated post‐therapy and correlated with reduced survival, highlighting TTK as a breast cancer biomarker and possible therapeutic target.

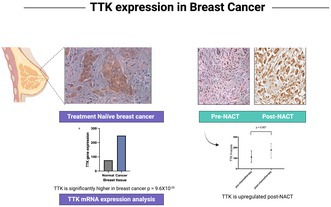

**Abbreviations**
BC: breast cancer
GEO: the gene expression Omnibus microarray database
hMPS1: Human protein kinase monopolar spindle 1
MCPH1: autosomal recessive primary microcephaly 1
NACT: neoadjuvant chemotherapy
OS: overall survival
SAC: spindle assembly checkpoint
TNBC: triple negative breast cancer
TPBC: triple positive breast cancer
TTK: Threonine and tyrosine kinase

## introduction

Breast cancer (BC) is a frequently diagnosed malignancy in women, representing 11.7% of all reported cancer cases worldwide, according to data from the Global Cancer Observatory (GCO) in 2020 [1, 2, 3]. This incidence is anticipated to increase to 46% by 2040 [3]. Due to the heterogeneous nature of BC and its histological variety between individuals and within tumours, identifying agents that could aid in BC treatment is necessary [2]. Triple‐negative breast cancer (TNBC) is associated with poor prognosis and a propensity to metastasise, particularly to the brain and lungs [4]. Treatments of patients with TNBC are challenging due to the absence of molecular targets for therapy, which makes its relapse time fewer than 5 years after diagnosis [4, 5]. Neoadjuvant chemotherapy (NACT) has been widely administered to patients prior to surgery to increase survival outcomes [6, 7]. Treatment responses may vary between patients, and some patients might benefit more than others due to differences in tumour size between patient cohorts [8]. Thus, there is a pressing need to discover surrogate markers that can be utilised to accurately measure the response of BC to NACT therapy. Two significant biomarkers implicated in BC are threonine and tyrosine kinase (TTK) and autosomal recessive primary microcephaly 1 (MCPH1).

Human protein kinase monopolar spindle 1 (hMPS1), also known as TTK, is a dual serine/threonine kinase that plays a role in cell proliferation and the spindle assembly checkpoint (SAC) [9, 10 SAC initiates a signalling cascade that prevents premature chromosome segregation during mitosis [11, 12]. TTK is also involved in cytokinesis post‐mitosis [13, 14, 15] and its expression is highly regulated during cell cycle progression, with its levels peaking at the G2/M phase [14, 15]. TTK overexpression has been reported in different tumour types, including BC [14, 15, 16]. Recent studies have suggested that TTK is a potential therapeutic target in BC due to its role in controlling cell cycle progression and its association with chromosomal instability and aneuploidy [15, 17]. In TNBC subtypes, high mRNA expression levels of TTK kinase have been reported compared to other BC subgroups [14, 15]. TTK overexpression is significantly associated with reduced overall survival (OS) in patients with BC, as well as with cancer progression and poor prognosis in the triple‐positive BC (TPBC) subtype [15, 18, 19]. Moreover, MCPH1, also known as BRCT‐inhibitor of hTERT expression (BRIT1), is expressed in different tissues and localises to the nucleus [20, 21]. Microcephaly 1 (MCPH1) plays diverse roles in cell cycle ‐checkpoint and DNA repair regulation, and its mutation has been found to lead to genomic instability and cancer development [22, 23, 24, 25]. MCPH1 plays a significant role in BC, with reported reduced levels in 17 of 54 cases (32%) of BC cell lines, suggesting its role as a tumour suppressor gene [22]. Moreover, MCPH1 levels were depleted in 93 of 319 cases (29%) of BC associated with high tumour grade and TNBC phenotype and identified as an independent predictor of overall BC‐specific survival [24].

**Figure 1:**
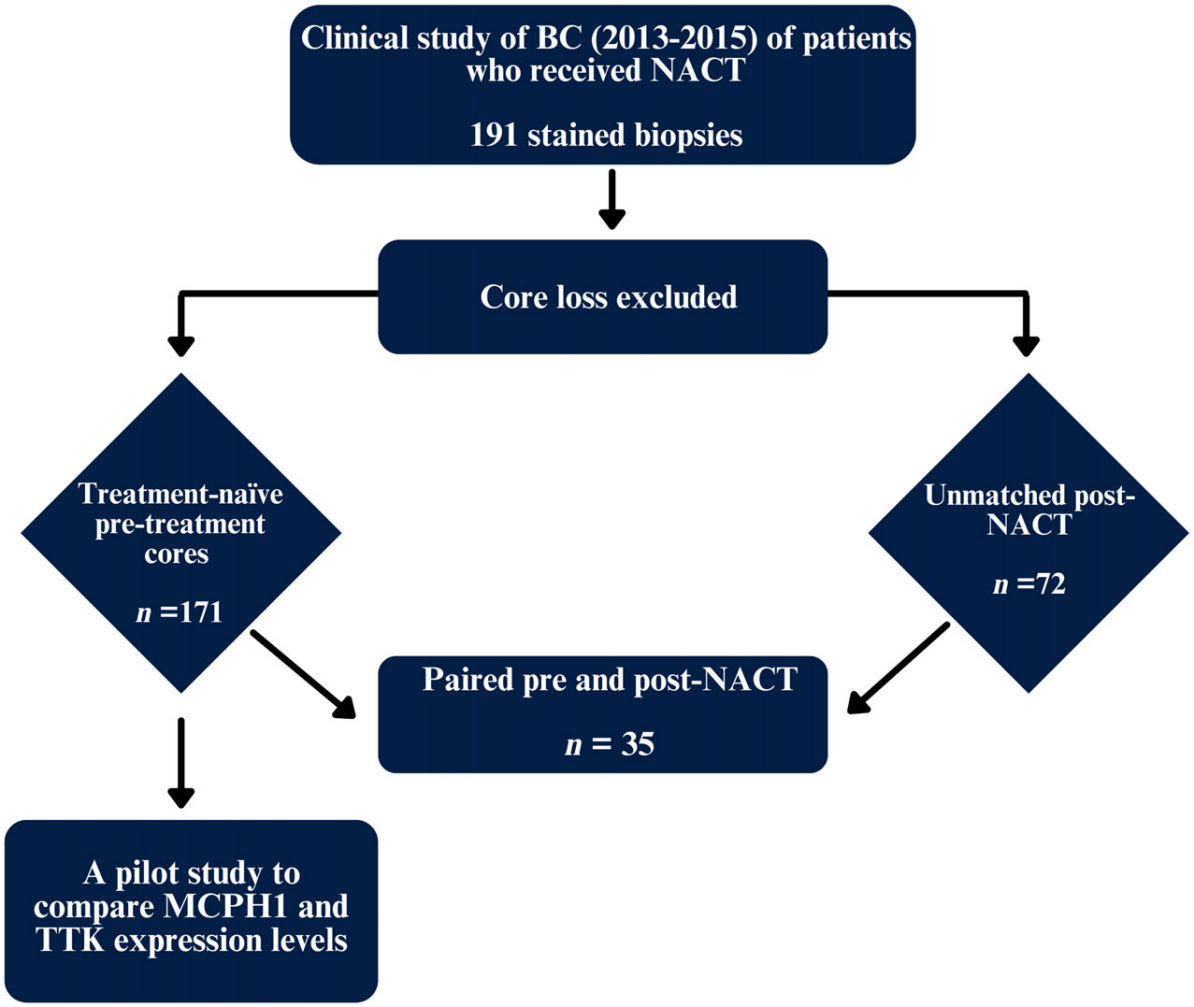
Flow diagram of the study showing 191 BC cases were reviewed; 171 treatment‐naïve pre‐treatment cores and 72 unmatched post‐neoadjuvant chemotherapy (NACT) were evaluated for threonine and tyrosine kinase (TTK) expression after excluding damaged cores. Thereafter, 35 paired pre‐ and post‐NACT cases were correlated for TTK expression. A pilot study was performed to correlate the expression of TTK and microcephaly 1 (MCPH1) in the pre‐NACT cohort. [Colour figure can be viewed at wileyonlinelibrary.com]

This study investigated TTK protein expression in BC biopsies before and after administration of NACT to determine its role as a potential biomarker and therapeutic target in BC. The BC cohort consisted of 171 treatment‐naïve pre‐treatment cores (unmatched pre‐NACT) and 72 post‐NACT unmatched samples (Figure 1). Moreover, TTK expression was investigated in a small cohort of 35 paired pre‐ and post‐NACT samples. Previous work by our group showed decreased MCPH1 protein expression in BC tissue [26]. In the present study, we investigate the relationship between the expression of TTK and MCPH1 in 42 BC pre‐NACT samples [24, 26]. Correlated to BC patient survival, we show that TTK is a potential biomarker for BC and a therapeutic target in MCPH1‐deficient BC cells.

## Materials and methods

### Patient samples

A cohort of BC tissue from NACT‐treated patients at Leeds Teaching Hospitals NHS Trust (LTHT) between 1 January 2005 and 30 April 2013, followed by breast surgery, was studied. The patient cohort consisted of primary and operable invasive carcinomas (including inflammatory BC) which were identified using the LTHT database. A limited number of matched pre‐ and post‐NACT samples (35) were available for study due to a lack of tissue or clinicopathological data. The NACT regimen administered to patients consisted of epirubicin, cisplatin and fluorouracil (5‐FU) (ECF) or fluorouracil, epirubicin and cyclophosphamide (FEC).

### The Gene Expression Omnibus (GEO) microarray database

TTK gene expression and OS were assessed in 1402 BC patients with available clinical data using the Kaplan–Meier (KM) plotter tool. The gene expression data were obtained from the GEO (Affymetrix HGU133A and HGU133 + 2 microarrays), the European Genome–phenome Archive (EGA) and The Cancer Genome Atlas (TCGA), which included cases of positive molecular biomarkers [oestrogen receptor (ER), progesterone receptor (PR) and human epidermal growth factor 2 (HER2)] and multiple clinicopathological parameters, including tumour grade (grades 1, 2 and 3), lymph node status, TP53 status (mutated and wild‐type) and intrinsic subtypes (basal, luminal A, luminal B and HER2‐positive).

### Immunohistochemical (IHC) staining and analysis

A mouse monoclonal antibody raised against TTK (TTK D‐8; Santa Cruz, Dallas, TX, USA) and a rabbit polyclonal MCPH1 antibody (ab2612; Abcam, Cambridge, UK) were optimised under different conditions on cell lines and on TMA slides (data are found in the Supporting information, Appendix, Figure S1). The formalin‐fixed paraffin‐embedded (FFPE) slides and TMA were deparaffinised in xylene, rehydrated in graded ethanol and washed in running water. Endogenous peroxidase activity was blocked using hydrogen peroxide (H_2_O_2_) buffer (BLOXALL; Vector Labs, Kirtlington, UK) for 20 min and washed in Tris‐buffered saline (TBS) for 5 min. For TTK staining, antigen retrieval was performed with 10 mM sodium citrate buffer (pH 6.0) for 20 min and MCPH1 staining with 10 mM citric acid buffer (pH 6.0) for 4 min in a microwave on full power. A 1:10 casein solution (Vector Labs) was used to block non‐specific binding, and the samples were incubated for 20 min in a moist chamber. The sections were incubated overnight at 4°C with 1:100 TTK or 1:100 MCPH1 antibody. Secondary staining was performed using a Novolink Polymer Kit (Leica Biosystems, Newcastle upon Tyne, UK) according to the manufacturer's protocol and counterstained using haematoxylin. Cytoplasmic TTK staining was scored based on the intensity of the staining 0, 1+, 2+ and 3+ corresponding to the presence of negative, weak, moderate to very strong staining, respectively. The percentage of cells for each staining intensity was scored and used to determine the H‐score using the following formula: H‐score = (% tumour cells weakly stained 1 × 1) + (% tumour cells moderately stained 2 × 2) + (% tumour cells at strongly stained 3 × 3). A score between 0 and 300 was obtained, where 300 was the strongest positive stain (representing 100%). Inflammatory cells, blood vessels and normal breast cells were excluded from scoring. A clinically relevant cut‐off point of 77.5 was generated using receiver operating characteristic (ROC) curve analysis software (data are found in the Supporting information, Appendix, Figure S2) [27].

MCPH1 nuclear staining was scored by S.M.B. and A.A.A. as a percentage of the positive cells in relation to the total number of tumour cells present. TMA slides were scanned using a high‐resolution digital scan scope T3 scanner (Aperio Technologies, Swindon, UK) at 40× magnification, then duplicate cores were scored and the mean value was calculated. Based on previous BC studies, a cut‐off point of 35% was used to dichotomised nuclear MCPH1 staining into high and low expression [24, 26].

### Statistical analysis

The statistical analyses were performed using the Statistical Package for the Social Sciences (SPSS), version 29 (IBM Corp., Armonk, NY, USA). Normal distribution of data was initially tested. Continuous data analyses were performed using Pearson's test, while categorical variables with dichotomised data employed Spearman's or χ^2^ tests for comparison of variables in pre‐ and post‐NACT BC cohorts. All statistical tests and comparisons were two‐sided, with a significance level set at *P* ≤ 0.05. In order to allow for multiple testing, a Benjamini–Hochberg correction was applied to adjust *P*‐values. OS was performed using KM, with the log‐rank test to assess statistical significance. Evaluation of the correlation between changes in the expression of the matched paired pre‐ and post‐NACT was carried out using the Wilcoxon signed‐rank test.

## Results

### TTK mRNA is up‐regulated in BC and correlates with reduced overall survival

**Figure 2:**
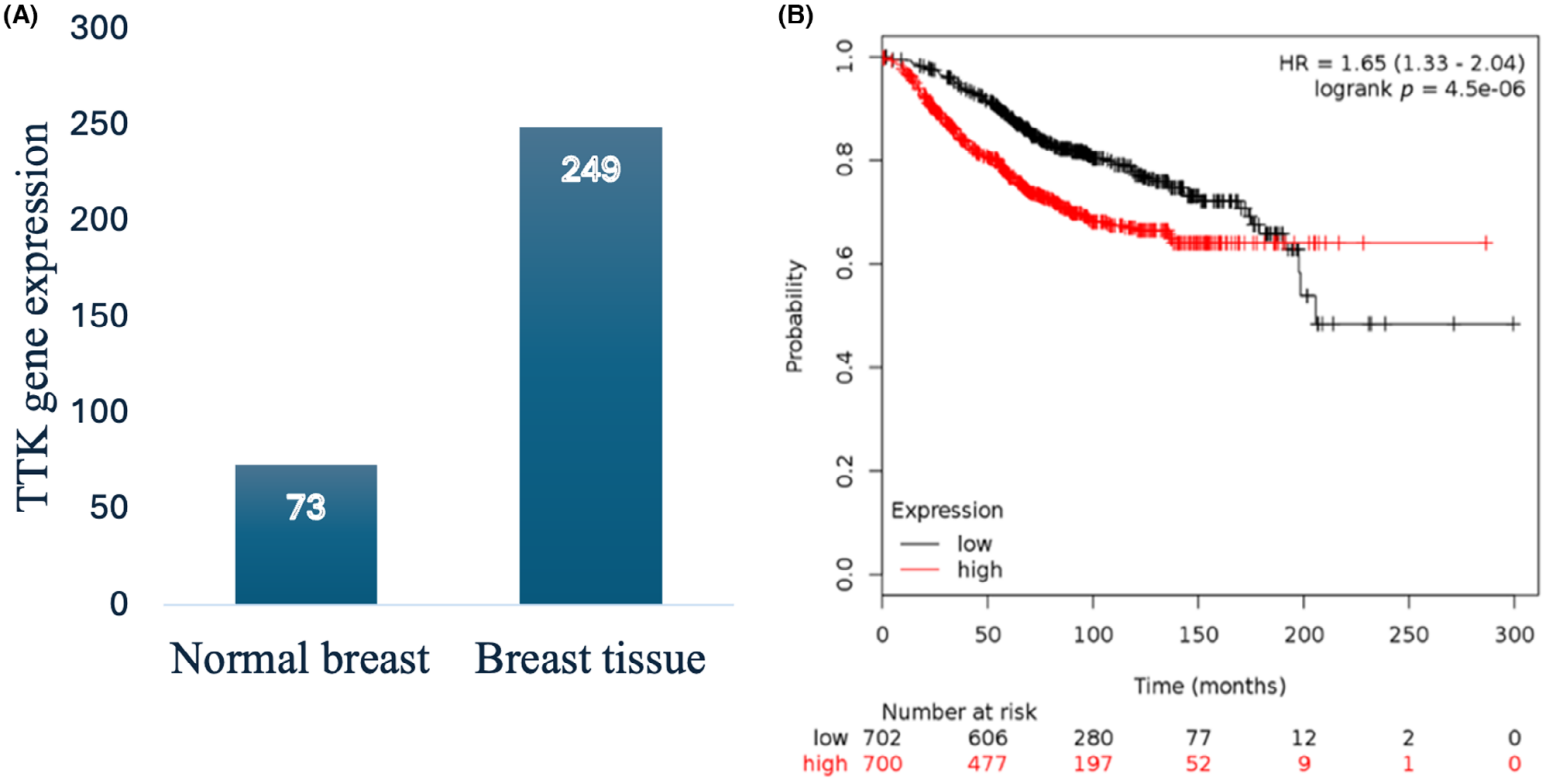
Threonine and tyrosine kinase (TTK) mRNA expression in breast samples. (A) TTK mRNA expression is increased 3.4‐fold in breast cancer (BC) samples compared to normal tissue (*P* = 9.6 × 10^−26^). (B) Kaplan–Meier plotter analysis of TTK mRNA expression in 1402 BC patients showing reduced overall survival with increased expression (*P* = 4.5 × 10^−6^). [Colour figure can be viewed at wileyonlinelibrary.com]

TTK mRNA expression analysis revealed elevated levels in BC tissue compared to normal breast tissue (*P* = 9.6 × 10^−26^). The patient data were split into two groups according to the median value of TTK mRNA expression. The median TTK mRNA expression in BC (*n* = 6547 patients) was 249, while the median in normal breast tissue (*n* = 76 patients) was 73, revealing a 3. 4‐fold increase in BC compared to normal breast tissue (Figure 2A). TTK mRNA expression was assessed in 1402 BC patients with available clinical data based on OS using the online KM tool [28]. The results showed a significant correlation between high TTK mRNA expression and reduced OS in the BC cohort (*P* = 4.5 × 10^−6^) (Figure 2B).

### Clinicopathological parameters and survival data

Clinicopathological characteristics, including age, tumour size, grade, lymph node (LN) status, the Nottingham Prognostic Index (NPI) and inflammation status, pathological response for the treatment‐naïve pre‐treatment cores and post‐NACT cohorts were combined and are shown in Table 1. The sample was divided by age into ≥ 50 and < 50 years based on menopausal age, with the patient's median age being 48 years. The patient's median follow‐up OS was 46.5 months (range = 8–96 months). The clinicopathological characteristics of the matched paired pre‐ and post‐NACT cohorts are presented in Table 2. The patient's median OS rate was 63 months. The most common histological type was invasive ductal carcinoma of no special type.

**Table 1:** Clinicopathological characteristics in the treatment naïve of pre‐treatment cores and post‐NACT cohort

Characteristics	Frequency (%)	
	Pre‐NACT	Post‐NACT
	*n* = 171	*n* = 72
Age characteristics	*n* = 169	
Age median	48	46
Range (years)	23–88	22–72
Age distribution (years)		NA
≤ 50	102 (60.4%)	
> 50	67 (39.6%)	
Tumour size		
Size distribution (mm)		*n* = 72
< 30	NA	30 (41.7)
≥ 30		42 (58.3)
Tumour grade	*n* = 163	*n* = 71
G1	11 (6.7%)	6 (8.5%)
G2	66 (40.5%)	32 (45.5%)
G3	86 (52.8%)	32 (45.5%)
Unknown	–	1 (NA)
Lymph node status		*n* = 72
LN 0	NA	29 (40.5%)
LN 1		19 (26%)
LN 2		13 (17.3%)
LN 3		8 (11.5%)
Unknown		3 (4.3%)
Inflammatory	*n* = 74	
Yes	18 (24.3%)	NA
No	56 (75.7%)	
Histological tumour type	*n* = 170	NA
Invasive ductal carcinoma	145 (85.3%)	
Other special type	25 (14.7%)	
Unknown	–	
Pathological response		*n* = 72
Partial	NA	51 (56.7%)
Complete		7 (7.8%)
None		14 (15.6%)
Metastases	NA	*n* = 69
Yes		26 (37.9%)
No		43 (62.1%)
Deceased	NA	*n* = 71
Yes		15 (20.5%)
No		56 (79.4%)
Follow‐up overall survival/months	NA	*n* = 72
Mean		46.53
Median		42.50
Range		(8–96)
Nottingham Prognostic Index	NA	*n* = 64
Good		1 (18%)
Moderate		23 (36%)
Poor		29 (45%)

NACT, neoadjuvant chemotherapy; *n*, number of patient samples; NA, not available.

**Table 2:** Clinicopathological characteristics for matched paired pre‐ and post‐NACT

Characteristics	Frequency (%)	
*n* = 35	Pre‐NACT	Post‐NACT
Age (years)	*n* = 33	NA
Median	46	
Range	23‐72	
Age distribution		NA
< 50	27 (79.4)	
> 50	7 (20.6)	
Tumour size (mm)		*n* = 35
Mean	NA	1.9
Range		1–4
Size distribution (mm)		*n* = 35
< 30	NA	13 (37.1)
≥ 30		22 (62.9)
Tumour grade on pre‐core	*n* = 35	
G1	4 (11.8)	4 (11.8)
G2	15 (44.1)	15 (44.1)
G3	15 (44.1)	15 (44.1)
Unknown	–	
Lymph node status		*n* = 35
LN 0	NA	13 (38.2)
LN 1		11 (32.4)
LN 2		7 (20.6)
LN 3		3 (8.8)
Inflammatory	*n* = 35	
Yes	7 (20.6)	NA
No	27 (79.4)	
Histological tumour type on pre‐core	*n* = 35	NA
Invasive ductal carcinoma (IDC)	31 (88.6)	
Other special type	2 (5.7)	
Unknown	2 (5.7)	
Pathological response		*n* = 35
Partial	NA	29 (82.9)
Complete		0 (0)
None		6 (17.1)
Metastases	NA	*n* = 35
Yes		11 (32.4)
No		23 (67.6)
Deceased	NA	*n* = 35
Yes		8 (27.6)
No		21 (72.4)
Follow‐up overall survival/months	NA	*n* = 35
Mean		77
Median		63
Range		8–153

NACT, neoadjuvant chemotherapy; *n*, number of patient samples; NA, not available.

### TTK protein expression analysis, cut‐off point determination and expression in paired pre‐ and post‐NACT cohort

**Figure 3:**
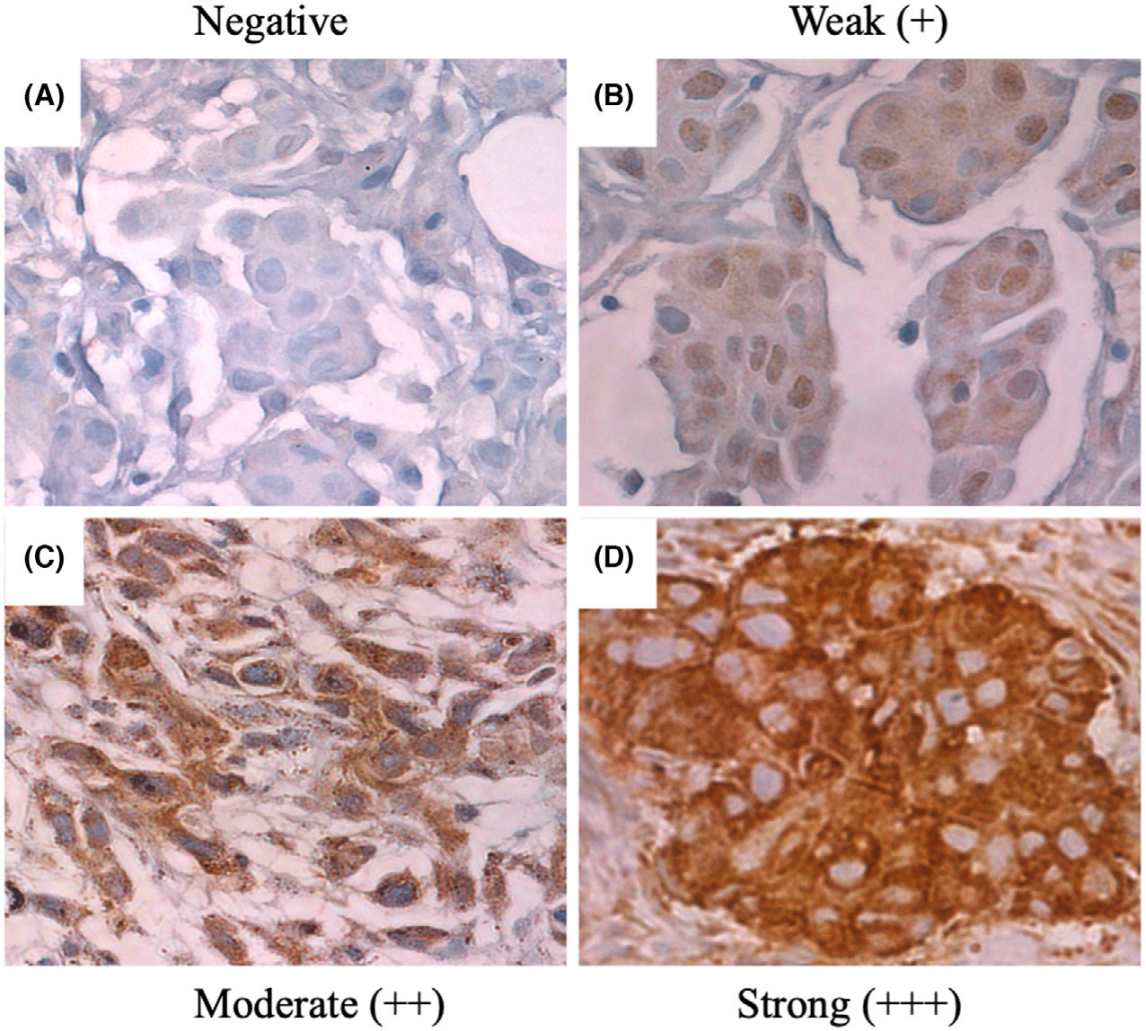
Threonine and tyrosine kinase (TTK) protein expression staining using immunohistochemistry in the pre‐neoadjuvant chemotherapy (NACT) cohort. Representative images of TTK staining (cytoplasmic). (A) Negative TTK cytoplasmic staining; (B) weak (1+) TTK cytoplasmic staining; (C) moderate (2+) TTK cytoplasmic staining; (D) strong (3+) TTK cytoplasmic staining.

TTK cytoplasmic/membranous staining was evaluated in the treatment‐naïve pre‐treatment cores and post‐NACT and in the paired pre‐ and post‐NACT cohorts using IHC (Figure 3). Staining intensities were scored as negative, weak, moderate or strong in BC tissue samples using the H‐scoring system. In the treatment‐naïve pre‐treatment cohort, dichotomous analysis of TTK expression levels showed high TTK expression (> cut‐off point) in 39.6% (67 of 169) cases, while 60.4% (102 of 169) cases showed low (< cut‐off point) TTK expression. In the post‐NACT cohort, high TTK expression was observed in 86% (59 of 69), while 14% (10 of 69) cases showed low TTK expression, suggesting a possible up‐regulation post‐NACT.

**Figure 4:**
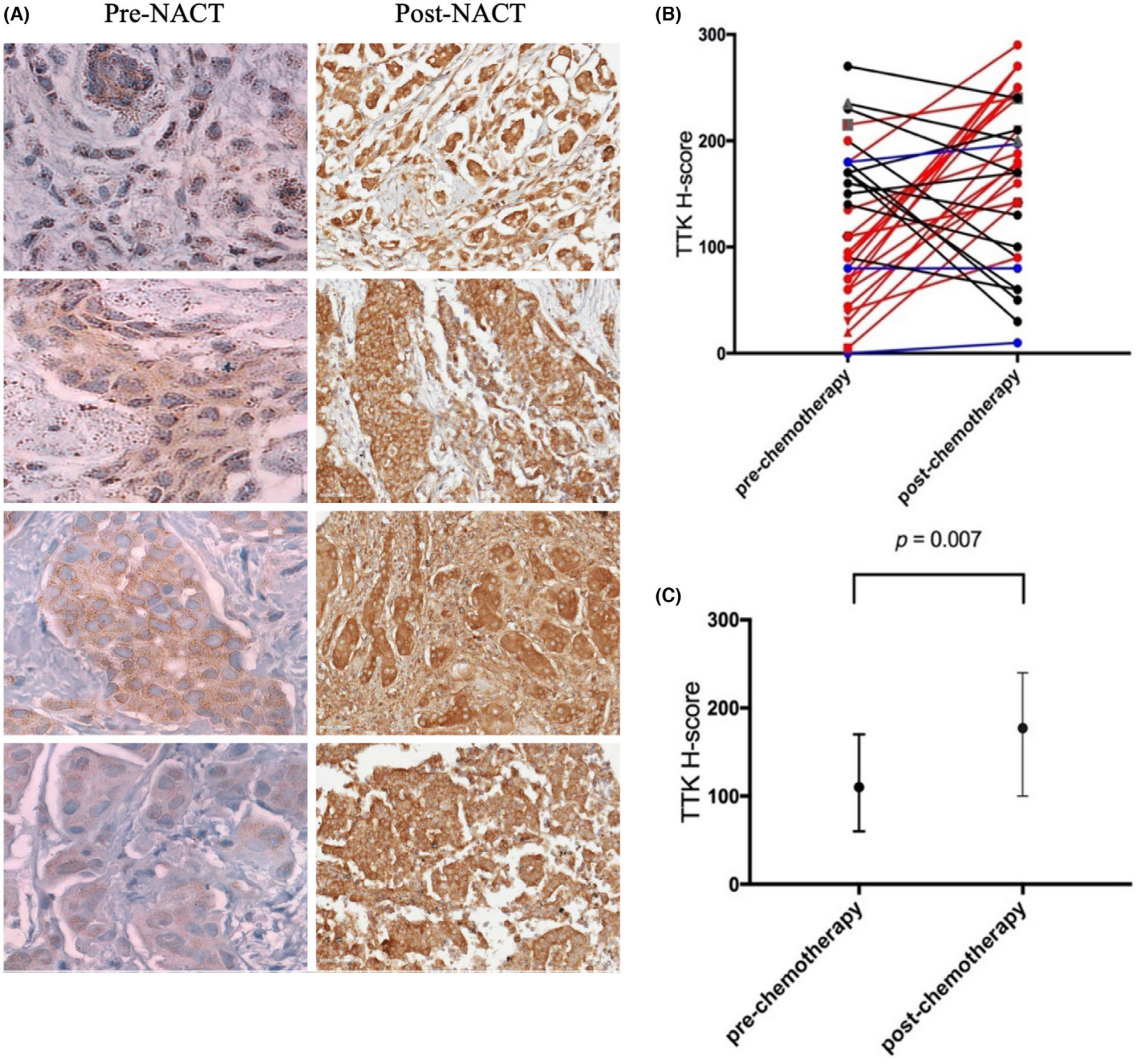
Threonine and tyrosine kinase (TTK) protein expression staining using immunohistochemistry in the paired pre‐ and post‐neoadjuvant (NACT) breast cancer cohort. (A) Staining represents the significant up‐regulation of TTK expression in breast cancer samples after NACT treatment. (B) Expression levels in paired samples are linked by lines; red lines indicate up‐regulation, black lines indicate down‐regulation, blue lines indicate no change in expression. (C) Shows median H‐score of TTK expression.

However, in the paired pre‐ and post‐NACT (*n* = 35), 57% (20 of 35) cases showed a statistically significant increase in the median H‐score post‐NACT, suggesting a possible up‐regulation post‐NACT (Wilcoxon's signed‐rank test, *P* = 0.007) (Figure 4).

### Correlation between TTK protein expression and clinicopathological parameters

Correlation between TTK expression and clinicopathological parameters was performed on treatment‐naïve pre‐treatment cores, unmatched post‐NACT cohort, and paired pre‐ and post‐NACT cohorts. In the treatment‐naïve pre‐treatment cores (*n* = 171) there was no correlation between TTK expression and various clinicopathological features, such as age and tumour grade (Table 3). However, analysis of molecular biomarkers, including TNBC, using both continuous and categorical data (low and high TTK expression), did not reveal a correlation with TTK expression. HER2‐negative cases comprised 76.4%, while positive cases were 23.6%, and there was no correlation between TTK‐positive tumours and HER2‐negative subtypes (*P* = 0.666, Spearman's correlation coefficient = 0.867).

**Table 3:** Correlation between TTK expression with clinicopathological features in the treatment‐naïve pretreatment cores

Parameters	Continuous		Dichotomous				
	Frequency (%)	^d^ *P*‐value	High *n* (%)	Low *n* (%)	Median	Q1–Q3	^d^ *P*‐value
Tumour grade	*n* = 163	0.978^b^			3	2–3	0.980^c^
G1	6.7%		10 (90.9)	1 (9.1)			
G2	40.5%		42 (63.6)	24 (36.4)			
G3	52.8%		60 (69.8)	26 (30.2)			
Histological type	*n* = 122	0.756^b^					0.867^c^
IDC	68.0%		98 (67.6)	47 (32.4)			
Other special types	28.7%		18 (NA)	7 (NA)			
Age (years)	*n* = 169	0.837^a^			97.50	60–165	0.867^c^
< 50	(60.4)		71 (69.6)	31 (30.4)			
> 50	39.6%		44 (65.7)	23 (34.3)			
Inflammatory	*n* = 74	0.837^b^			NA		0.867^c^
Yes	24.3%		14 (77.8)	4 (22.2)			
No	75.7%		36 (64.3)	20 (35.7)			
ER	*n* = 168	0.837^b^			6	0–8	0.867^c^
Negative	36.9%		44 (71)	18 (29)			
Positive	63.1%		70 (66)	36 (34)			
PR	*n* = 167	0.837^b^			0	0–7	0.974^c^
Negative	50.9%		57 (67.1)	28 (32.9)			
Positive	49.1%		56 (68.3)	26 (31.7)			
HER2	*n* = 157	0.666^b^			1	0–1	0.867^c^
Negative	76.4%		84 (70)	36 (30)			
Positive	23.6%		21 (56.8)	16 (43.2)			
Triple‐negative breast cancer	*n* = 169	0.846^b^			NA		0.867^c^
Negative	75.7%		86 (67.2)	42 (32.8)			
Positive	24.3%		29 (70.7)	12 (29.3)			
Ki67	*n* = 59	0.978^b^			50	20–80	0.867^c^
Negative	20.3%		7 (58.3)	5 (41.7)			
Positive	79.7%		32 (68.1)	15 (31.9)			

Continuous and dichotomous data were utilised. TTK, threonine and tyrosine kinase; ER, oestrogen receptor; PR, progesterone receptor; HER2, human epidermal growth factor receptor 2; IDC, invasive ductal carcinoma; IDC, invasive ductal carcinoma; NA, not available.

^a^ Independent *t‐*test;

^b^ Pearson's correlation;

^c^ Spearman's correlation;

^d^ Benjamini–Hochberg adjusted *P*‐values.

Moreover, in the unmatched post‐NACT cohort (*n* = 72), dichotomised data analysis showed no correlation between high TTK expression and the TNBC subtype (*P* = 0.975, Spearman's correlation coefficient = 0.105) or metastasis (*P* = 0.975, Spearman's correlation coefficient = 0.105) (Table 4). Continuous analysis showed no correlation with LN status (*P* = 0.494). Furthermore, in the paired pre‐ and post‐NACT cohort (*n* = 35), continuous data analysis revealed a significant correlation between TTK expression and LN status (*P* = 0.026) (Table 5). However, no correlation was found between TTK expression and other clinicopathological parameters or molecular biomarkers.

**Table 4:** Correlation between TTK expression with clinicopathological features in the unmatched post‐NACT cohort

Parameters	Continuous		High *n* (%)	Low *n* (%)	Dichotomous		
	Frequency (%)	^d^ *P‐*value			Median	Q1–Q3	^d^ *P‐*value
Tumour grade	*n* = 71	0.975^b^			2	2–3	0.477^c^
G1	8.5%		5 (100)	0 (0)			
G2	45.1%		27 (87.1)	4 (12.9)			
G3	46.5%		25 (80.6)	6 (19.4)			
Age (years)	*n* = 72	0.975^a^			46	40. 25–51.50	0.765^c^
< 50	72.2%		44 (86.3)	7 (13.7)			
> 50	27.8%		15 (83.3)	3 (16.7)			
Inflammatory	*n* = 72	0.975^b^			NA		0.765^c^
Yes	16.7%		9 (81.8)	2 (18.2)			
No	83.3%		50 (86.2)	8 (13.8)			
ER	*n* = 66	0.975^b^			4.50	0–8	0.403^c^
Negative	31.8%		15 (75)	5 (25)			
Positive	68.2%		39 (90.7)	4 (9.3)			
PR	*n* = 66	0.975^b^			0	0–6.25	0.375^c^
Negative	56.1%		29 (80.6)	7 (19.4)			
Positive	43.9%		25 (92.6)	2 (7.4)			
HER2	*n* = 67	0.975^b^			0	0	0.373^c^
Negative	100%		55 (85.9)	9 (14.1)			
Positive	0%		0	0			
Triple‐negative breast cancer	*n* = 68	0.975^b^			NA		0.150 ^c^
Negative	76.5%		45 (91.8)	4 (8.2)			
Positive	23.5%		11 (68.8)	5 (31.3)			
Ki67	*n* = 53	0.975^b^			15	5–60	0.375^c^
Negative	54.7%		25 (89.3)	3 (10.7)			
Positive	45.3%		18 (75.0)	6 (25)			
Tumour size (mm)	*n* = 72	0.975^b^			33.50	20–50	0.283^c^
< 30	41.7%		23 (82.1)	5 (17.9)			
> 30	58.3%		36 (87.8)	5 (12.2)			
Nottingham Prognostic Index	*n* = 64	0.975^b^			NA		0.689^c^
Good	18.8%		10 (90.9)	1 (9.1)			
Moderate	35.9%		18 (81.8)	4 (18.2)			
Poor	45.3%		23 (82.1)	5 (17.9)			
Lymph node status	*n* = 51	0.494^b^					0.283^c^
N0	40.3%		23 (82.1)	5 (17.9)	1	0–2	
N1	26.4%		15 (83.3)	3 (16.7)			
N2	18.1%		12 (100)	0 (0)			
N3	11.1%		6 (75)	2 (25)			
N4	4.2%		3 (100)	0 (0)			
Pathological response	*n* = 69	0.975			NA		0.689^c^
Partial	70.8%		41 (85.4)	7 (14.6)			
Complete	9.7%		6 (85.7)	1 (14.3)			
None	19.4%		12 (85.7)	2 (14.3)			
Metastases	*n* = 70	0.975^b^			NA		0.150^c^
No	62.3%		38 (92.7)	3 (7.3)			
Yes	37.7%		18 (72)	7 (28)			

Continuous and dichotomous analyses were utilised. TTK, threonine and tyrosine kinase; NACT, neoadjuvant chemotherapy; NA, not available; ER, oestrogen receptor; PR, progesterone receptor; HER2, human epidermal growth factor receptor 2.

^a^ Independent *t‐*test;

^b^ Pearson's correlation;

^c^ Spearman's correlation;

^d^ Benjamini–Hochberg adjusted *P*‐values.

**Table 5:** Correlation of TTK expression with clinicopathological parameters in the paired pre‐ and post‐NACT cohort

Parameters	Continuous							Dichotomised				
	Frequency (%)	^d^ *P*‐value	Frequency (%)	*P*‐value	High	Low	^d^ *P*‐value	High	Low	Median	Q1–Q3	^d^ *P*‐value
	Pre		Post		Pre			Post				
Tumour grade	*n* = 34	0.776^b^	*n* = 34	0.411^b^						2	2–3	0.774^c^
G1	11.8%		11.8%		4	0	0.440	4	0			
G2	44.1%		44.1%		9	6		13	2			
G3	44.1%		44.1%		11	4		12	3			
Age	*n* = 34	0.916^a^	*n* = 34	0.690^a^			0.958			46	40.75–49	0.774^c^
< 50	79.4%		79.4%		18	9		22	5			
> 50	20.6%		20.6%		5	2		7	0			
Inflammatory	*n* = 34	0.776^b^	*n* = 34	0.420^b^			0.958			NA		0.973^c^
Yes	20.6%		20.6%		19	8		23	4			
No	79.4%		79.4%		5	2		6	1			
ER	*n* = 35	0.916^b^		0.411^b^			0.958			6.50	0–8	0.576^c^
Negative	34.3%		29.4%		9	3		7	3			
Positive	65.6%		70.6%		1	8		22	2			
PR	*n* = 35	0.916^b^		0.549^b^			0.958			5	0–8	0.576^c^
Negative	40%		52.9%		7	4		14	4			
Positive	60%		47.1%		14	10		15	1			
HER2	*n* = 35	0.916^b^		0.651^b^			0.736			0	0–1	0.774^c^
Negative	65.7%		87.5%		14	9		24	4			
Positive	34.3%		12.5%		10	2		3	1			
TNBC	*n* = 35	0.916^b^		0.411^b^			0.958			NA		0.576^c^
Negative	74.3%		73.5%		18	8		23	2			
Positive	25.7%		26.5%		6	3		6	3			
Ki67	*n* = 31	0.916^b^		0.459^b^			0.958			40	20–80	0.576^c^
Negative	29%		15 (62.5)		7	6		13	2			
Positive	71%		9 (37.5)		16	6		6	3			
Tumour size (mm)	NA			0.411^b^		NA				40	22–60	
< 30			13 (37.1)					10	3			0.576^c^
> 30			22 (62.9)					20	2			
Nottingham Prognostic Index	NA			0.459^b^	NA	NA	NA					0.940^c^
Good			13 (38.1)					11	2			
Moderate			20 (58.8)					17	3			
Poor			1 (2.9)					20	0			
Lymph node status	NA		*n* = 34	0.026^b^	NA	NA	NA			NA		0.576^c^
N0			13 (38.2)					1	3			
N1			11 (32.4)					9	2			
N2			7 (20.6)					7	0			
N3			3 (8.8)					3	0			
N4			0					0	0			
Pathological response	NA		*n* = 34	0.651^b^	NA	NA	NA			NA		0.774^c^
Partial			29 (82.9)					24	5			
Complete			0					0	0			
None			5 (17.1)					5	0			
Metastases	NA		*n* = 34	0.651^b^	NA	NA	NA			NA		0.831^c^
No			23 (67.6)					20	3			
Yes			11 (32.4)					9	2			

TTK, threonine and tyrosine kinase; NA, not available; NACT, neoadjuvant chemotherapy; ER, oestrogen receptor; PR, progesterone receptor; HER2, human epidermal growth factor receptor 2. TNBC, triple negative breast cancer.

^a^ Independent *t‐*test;

^b^ Pearson's correlation;

^c^ Spearman's correlation;

^d^ Benjamini–Hochberg adjusted *P*‐values.

### Correlation of TTK protein expression and overall survival

**Figure 5:**
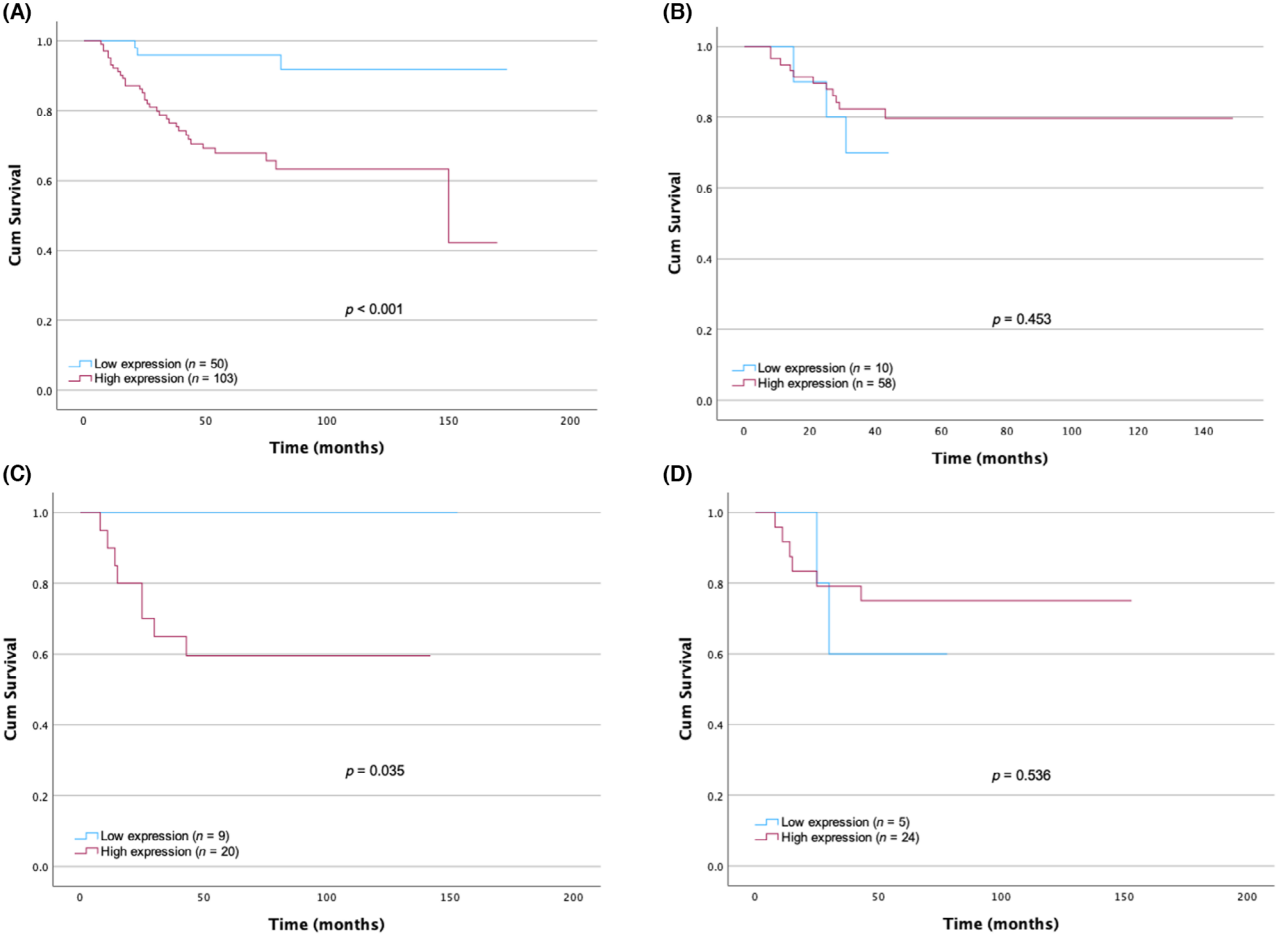
Kaplan–Meier survival analyses for threonine and tyrosine kinase (TTK) expression levels against overall survival (OS) (months) in the treatment‐naïve pre‐cores, paired and unmatched pre‐ and post‐neoadjuvant (NACT) cohorts. (A) TTK expression significantly correlates with OS in the treatment‐naïve pre‐treatment cohort. High TTK expression significantly predicted poorer survival (*P* < 0.001). (B) No significant correlation was detected in the unmatched post‐NACT. (C) TTK expression and OS are correlated in the paired pre‐NACT (*P* = 0.035). (D) No correlation between TTK expression and OS in the paired post‐NACT. [Colour figure can be viewed at wileyonlinelibrary.com]

KM survival analyses were performed to evaluate the correlation between OS and dichotomous TTK expression via ROC curve analysis, with an H‐score cut‐off point of 77.5. These analyses generated low and high TTK expression groups. In the treatment‐naïve pre‐treatment cores, KM survival analysis showed a significant correlation between TTK expression and OS (*P* < 0.0001) (Figure 5A). Low TTK expression (*n* = 50) predicted a better OS of 164 months, and high TTK expression (*n* = 103) predicted a poorer OS of 115 months. In the post‐NACT cohort, no correlation was seen between TTK expression and OS (Figure 5B). In the paired pre‐ and post‐NACT cohort, KM survival analysis revealed significant correlations between TTK expression and OS in the pre‐NACT cohort (*P* = 0.035) (Figure 5C). However, there was no significant correlation between survival and TTK expression in the matched‐paired post‐NACT cohort (*P* = 0.536) (Figure 5D).

### MCPH1 expression in the pre‐NACT BC cohort

**Figure 6:**
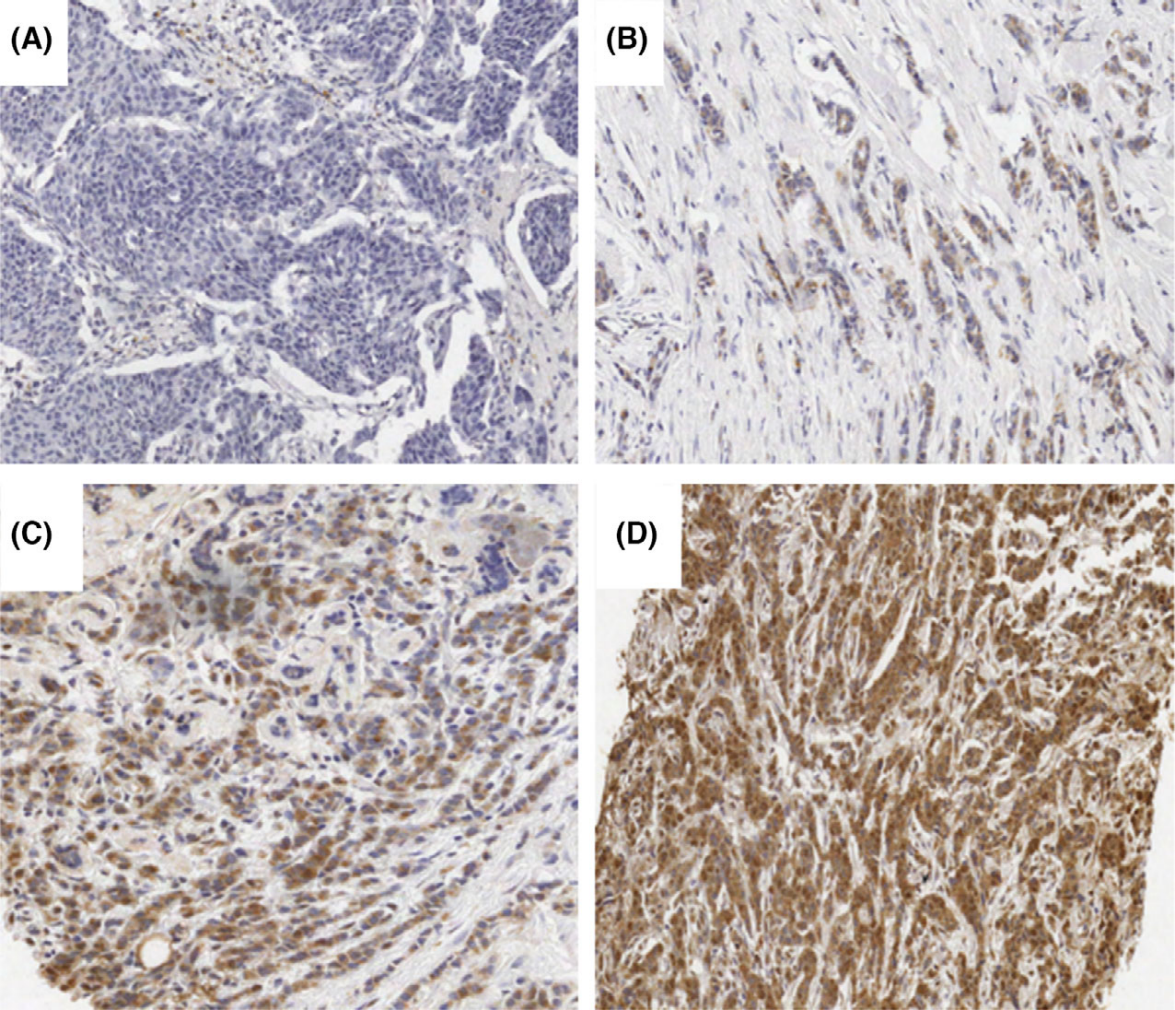
Microcephaly 1 (MCPH1) protein expression in BC core biopsies in pre‐neoadjuvant (NACT) samples using immunohistochemistry. Representative images of MCPH1 nuclear staining. (A) Negative, (B) weak (1+), (C) moderate (2+), (D) strong (3+) staining. [Colour figure can be viewed at wileyonlinelibrary.com]

MCPH1 protein expression was evaluated in the treatment‐naïve pre‐treatment cohort using IHC (Figure 6). Nuclear and cytoplasmic staining of MCPH1 was observed [24, 26]. MCPH1 staining intensities in pre‐NACT tumour samples, ranging from negative and weak to moderate and strong. Although cytoplasmic expression of MCPH1 was observed, it was excluded from this study due to significant background staining caused by the MCPH1 antibody. The percentage of tumour cells exhibiting positive nuclear MCPH1 staining was evaluated relative to the total number of tumour cells. The samples were dichotomised into low and high MCPH1 expression using a previously established cut‐off point of 35% [24, 26].

### Correlation analysis between TTK and MCPH1 expression

In a pilot study, 45 BC biopsies that had available scores for MCPH1 protein expression were stained for TTK. We aimed to evaluate TTK protein expression in patients with low‐protein expression levels of MCPH1 to validate the potential use of TTK inhibitors in MCPH1‐deficient BC patients. The overall median percentage of cells stained for MCPH1 was 40.8%, whereas the H‐score was 127.9 for TTK expression. Dichotomous data analysis revealed a significant correlation between MCPH1 and TTK expression (*P* = 0.020; Spearman's correlation). Furthermore, 24 of 45 (53.3%) of the patients had high TTK and high MCPH1 expression (Table 6). By contrast, nine of 45 (20%) patients had high TTK and low MCPH1 expression. Correlation analysis showed a significant correlation between TTK and MCPH1 expression (*P* = 0.0038).

**Table 6:** Correlation between TTK and MCPH1 expression in the pre‐NACT cohort

Correlation between TTK and MCPH1	Observed number of samples (%)	Expected number of samples (%)	*P*‐value
High TTK, high MCPH1	24 (53.3%)	19.8 (44%)	0.0038
High TTK, low MCPH1	9 (20%)	13.2 (29.3%)	
Low TTK, high MCPH1	3 (6.6%)	7.2 (16%)	
Low TTK, low MCPH1	9 (20%)	4.8 (10.6%)	

TTK, threonine and tyrosine kinase; MCPH1, microcephaly 1; NACT, neoadjuvant chemotherapy.

## Discussion

This study evaluates the gene and protein expression of TTK pre‐ and post‐administration of NACT. TTK mRNA expression analysis revealed significantly elevated levels in BC tissue compared to normal breast tissue, which suggests a potential role for TTK dysregulation in breast tumorigenesis, highlighting its relevance as a candidate biomarker for disease detection and prognosis [14, 15, 17]. TTK mRNA levels are lower in less proliferative cells and absent in the G0 phase cells, implying a possible relationship between high TTK levels and the cell proliferation rate during tumourigenesis induction [29, 30, 31]. Thus, the TTK checkpoint may compensate for other defects in the mitotic spindle checkpoint through overexpression during mitosis, protecting cancer cells from genomic instability and preventing cell death [14, 31]. Previous research has contributed to our understanding of TTK expression in TNBC and its implications, reporting elevated TTK levels and identifying unique molecular features associated with TTK dysregulation in TNBC, thereby highlighting its potential as a biomarker or therapeutic target in this subtype [14, 15, 30].

TTK, as a crucial regulator of the mitotic checkpoint, plays a pivotal role in ensuring accurate chromosome segregation during cell division [14, 15]. Dysregulation of TTK can lead to chromosomal instability, a hallmark of cancer, which may contribute to tumour progression and metastasis [17]. Elevated TTK expression may also enhance the proliferation and invasiveness of cancer cells, facilitating their spread to regional lymph nodes [29]. We did not observe an association between HER2 status and TTK‐positive tumours. This observation contradicts previous studies that observed a significant overexpression of TTK in TNBC compared to other subtypes characterised by high genomic instability [31]. Moreover, studies showed a significant correlation between TTK overexpression and HER2‐positive breast cancer [12, 14, 31]. However, no significant correlation was found between TTK expression and HER2 status, indicating that TTK plays a broader role in breast cancer progression, regardless of HER2 amplification [31]. The main differences between these studies lie in the chemotherapy regimen administered and the difference in sample size. The small size of paired samples in this study limits generalisability.

In line with our findings, previous research, such as the study by Xu *et al*. [12], also reported no significant association between TTK expression and LN status. This reinforces our observation that, while TTK serves as an independent predictor of disease‐free survival (DFS) and OS, its expression does not appear to directly influence LN involvement. These results highlight that TTK's prognostic value may be tied more to its role in tumour proliferation and aggressiveness rather than metastatic spread to the LNs [14, 19]. However, TTK expression and LN showed a positive correlation in the small paired pre‐ and post‐NACT cohorts which contradicts with the previous finding. This might be due to a limitation in the study of a small cohort.

The paired pre‐ and post‐NACT samples revealed a significant increase in TTK expression post‐chemotherapy in a substantial proportion of the cases. This finding suggests a potential role for TTK in mediating chemotherapy resistance or as a marker of treatment response, emphasising the need for further mechanistic studies to elucidate its implications in therapeutic resistance mechanisms. A number of TTK inhibitors have been developed and are currently in clinical use for different cancer types [19, 32, 33]. CFI‐402257 and NTRC 0066–0, inhibitors of TTK, show effective suppression of tumour growth *in vitro* and *in vivo* [15, 32]. Additionally, co‐administration of NTRC 0066–0 and docetaxel led to extended survival and tumour regression without causing toxicity in a mouse model of TNBC [15]. These studies suggest that TTK may serve as a potential therapeutic target for BC and that its inhibition may increase cell sensitivity to NACT and overcome resistance mechanisms [19, 32, 33].

Lower TTK expression was associated with better OS in the treatment‐naïve pre‐treatment cohort and in the matched‐paired pre‐NACT cohort, which is consistent with previous findings suggesting TTK as a favourable prognostic indicator in BC [14, 15, 31]. However, in the post‐NACT cohort, no significant correlation was observed between TTK expression and OS, indicating a potential alteration in the prognostic significance of TTK following chemotherapy. Further research is needed to elucidate the underlying mechanisms and clinical implications of TTK dysregulation in the context of NACT and its impact on survival outcomes in a larger sample size.

MCPH1 is expressed in several cancers, including BC [24, 34, 35]. MCPH1 nuclear staining was only considered in this study because variations in cytoplasmic staining levels were found using different MCPH1 antibodies [26]. MCPH1 has been identified as a novel hereditary BC susceptibility gene [36, 37, 38]. A recurrent heterozygous MCPH1 mutation c.904_916del was identified in 3.4% (five of 145) familial and 1.4% (16 of 1150) sporadic BC cases. High levels of chromosomal rearrangements were observed for carriers of *MCPH1* mutation, indicating that increased genomic instability and cancer development caused by MCPH1 haploinsufficiency [34, 36]. The function of MCPH1 as a BC susceptibility gene was supported by another study conducted to assess the expression of DNA damage proteins in familial and sporadic BC patients [37], although there is limited evidence relating to TTK and MCPH1 in BC. However, both TTK and MCPH1 have been shown to play a vital role in regulating BC cell progression and the homologous recombination pathway [14, 16, 22]. Studies indicate that high levels of TTK mRNA can help BC cells maintain proper chromosome segregation, which might protect them from further chromosomal imbalances. TTK has become a focus for potential BC therapies, especially as it is commonly overexpressed in HER2+ and TNBC cells compared to other types of BC and normal tissues [14, 15]. These findings suggest that TTK could be a valuable target for new treatments aimed at improving outcomes for BC patients [31, 32]. Moreover, given the role of MCPH1 in genomic instability and its role as a tumour suppressor gene, and the potential association between TTK and MCPH1 protein expression, our pilot study suggests that the group with low MCPH1 expression and high TTK expression might benefit from treatment with a synthetic lethal approach to develop novel treatments for MCPH1‐deficient BC patients [33]. To further confirm this finding, a larger cohort comparing both markers would enhance this conclusion regarding a potential association between TTK and MCPH1 expression and the potential to develop a treatment for MCPH 1‐deficient BC patients.

In conclusion, the findings presented highlight the significance of TTK expression in BC and its potential as a biomarker and therapeutic target. Continued research efforts aimed at unravelling the complexities of TTK biology hold promise for improving patient stratification, treatment outcomes and OS in BC. Targeting TTK pathways holds promise for BC treatment, as inhibiting TTK activity has shown efficacy in impairing tumour growth and enhancing chemotherapy sensitivity in pre‐clinical models.

## Conflicts of interest

The authors declare no conflicts of interest. The funders had no role in the design of the study; in the collection, analyses or interpretation of data, in the writing of the manuscript or in the decision to publish the results.

## Supporting information


**Figure S1:** Optimisation of TTK antibody on cell pellets and TMA cores using IHC. Multiple antibody dilutions (1/50 and 1/100) and antigen retrieval times (10 min and 20 min) using heat were performed to obtain the optimal TTK staining in HepG2, MCF‐7 cells and TMA. Images were taken using ×60.

**Figure S2:** Optimal cut‐off for TTK generated using ROC‐algorithm in cut‐off finder analysis. (a) Represent histogram showing cut‐off point (red line) which is 77.5 based on the H‐score of 171 core biopsies. (b) Showing ROC curve. AUC = area under the curve. The data is generated using the online cut‐off generator (http://molpath.charite.de/cutoff/index.jsp).

**Data S1:** Supporting Information.

## Data Availability

The data that support the findings of this study are available from the corresponding author upon reasonable request.
