# Supplementary figures and images for: Threonine and tyrosine kinase (TTK) mRNA and protein expression in breast cancer; prognostic significance in the neoadjuvant setting
Source: Histopathology. 2025 Jan 7;86(6):916–32. doi: 10.1111/his.15399 (PMC11964583; doi:10.1111/his.15399)

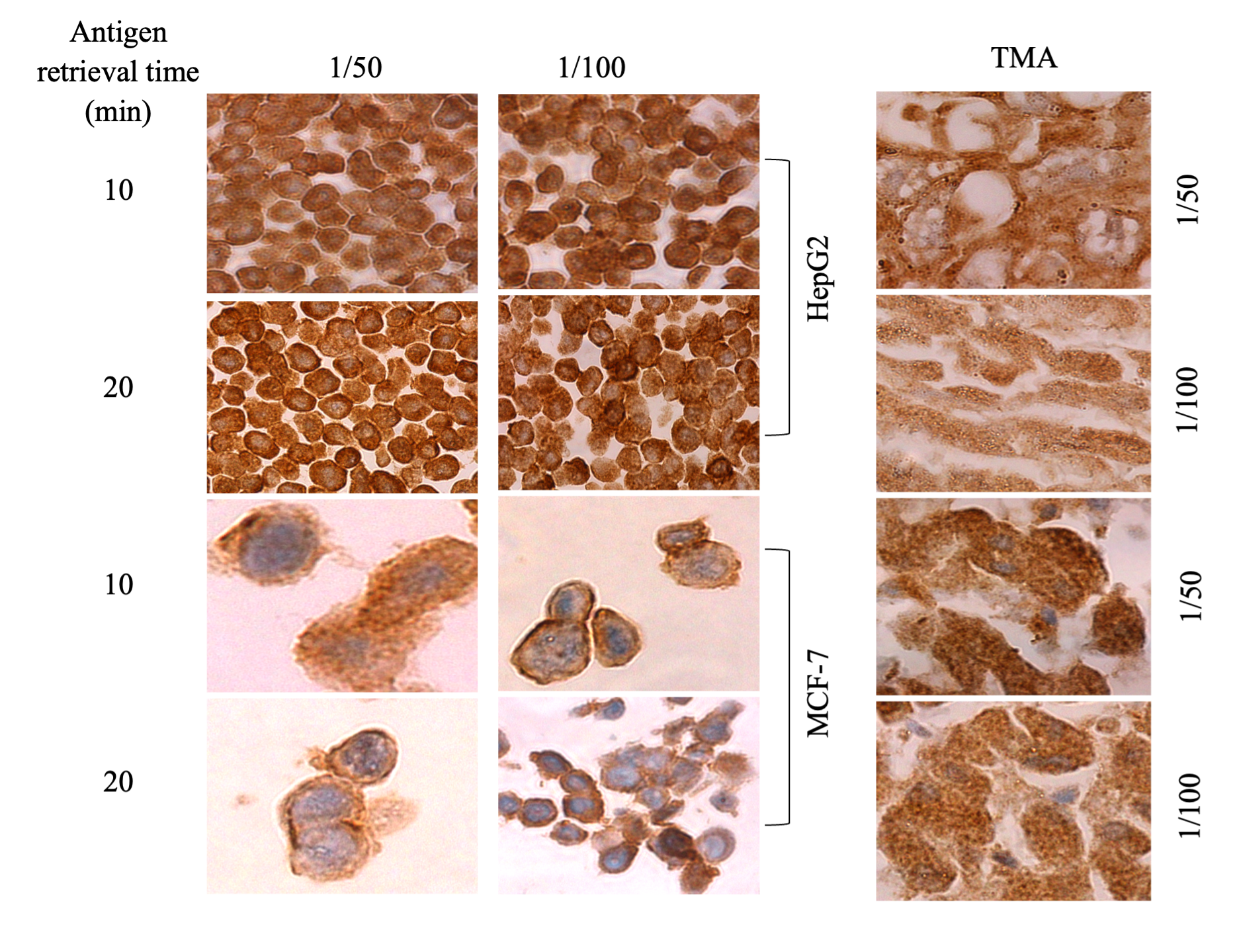

Supplement: Supplementary file 1 — Figure S1: Optimisation of TTK antibody on cell pellets and TMA cores using IHC. Multiple antibody dilutions (1/50 and 1/100) and antigen retrieval times (10 min and 20 min) using heat were performed to obtain the optimal TTK staining in HepG2, MCF‐7 cells and TMA. Images were taken using ×60. [file HIS-86-916-s003.tif]

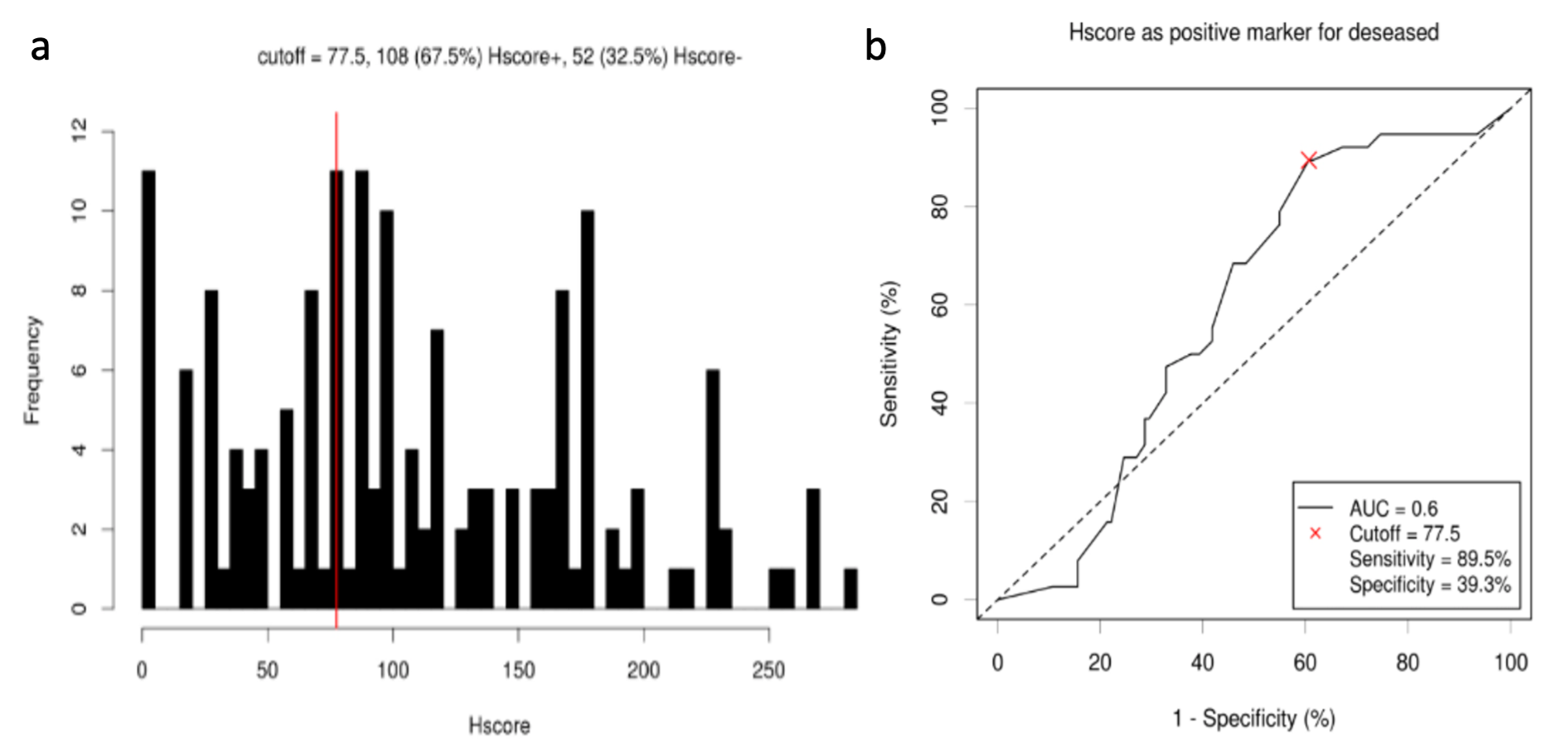

Supplement: Supplementary file 2 — Figure S2: Optimal cut‐off for TTK generated using ROC‐algorithm in cut‐off finder analysis. (a) Represent histogram showing cut‐off point (red line) which is 77.5 based on the H‐score of 171 core biopsies. (b) Showing ROC curve. AUC = area under the curve. The data is generated using the online cut‐off generator (http://molpath.charite.de/cutoff/index.jsp). [file HIS-86-916-s002.tif]
