# Supplementary material for: Threonine and tyrosine kinase (TTK) mRNA and protein expression in breast cancer; prognostic significance in the neoadjuvant setting
Source: Histopathology. 2025 Jan 7;86(6):916–32. doi: 10.1111/his.15399 (PMC11964583; doi:10.1111/his.15399)
Supplement: Supplementary file 3 — Data S1: Supporting Information. [file HIS-86-916-s001.docx]

**Appendix A**

Antigen retrieval time

(min)

10

20

10

20

1/50 1/100

HepG2

MCF-7

1/50

1/100

1/50

1/100


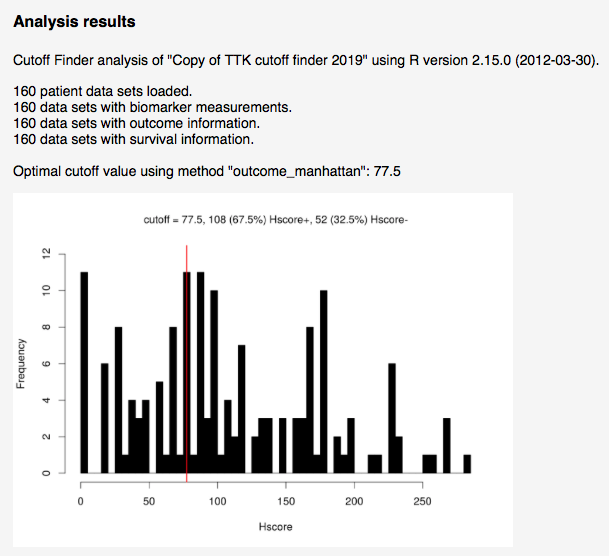

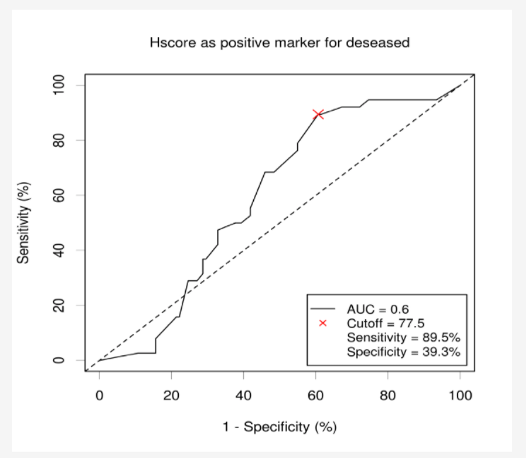


a

b

**Figure S2.** Optimal cut off for TTK generated using ROC-algorithm in cut-off finder analysis. a) Represents histogram showing cut-off point (red line) which is 77.5 based on the H-score of 171 core biopsies. b) Showing ROC curve. AUC = area under the curve. Data presented is generated using the online cut-off generator (http://molpath.charite.de/cutoff/index.jsp).

**Figure S1.** Optimization of TTK antibody on cell pellets and TMA cores using IHC. Multiple antibody dilutions (1/50 and 1/100) and antigen retrieval times (10 min and 20 min) using heat were performed to obtain the optimal TTK staining in HepG2, MCF-7 cells and TMA. Images were taken using X60.
